# Expression of concern: Excess mortality across countries in the Western World since the COVID-19 pandemic: ‘Our World in Data’ estimates of January 2020 to December 2022

**DOI:** 10.1136/bmjph-2023-000282eoc

**Published:** 2024-06-14

## Update and outcome to the journal’s expression of concern, August 2026

The journal has concluded its investigation into the concerns raised in the expression of concern below.

The article’s funding statement has been corrected^1^ to accurately reflect the nature of funding from the World Child Cancer - The Netherlands Foundation.

The article has also been retracted^2^ by the journal for misinformation in the discussion regarding the possible causes ofexcess mortality, and because the limited nature of the original work by the authors was not sufficiently described.

## Original expression of concern (14 June 2024)

The integrity team and editors are investigating issues raised regarding the quality and messaging of this work. The Princess Máxima Centre, which is listed as the affiliation of three of the four authors, is also investigating the scientific quality of this study. The integrity team has contacted the institution regarding their investigation.

Readers should also be alerted to misreporting and misunderstanding of the work. It has been claimed that the work implies a direct causal link between COVID-19 vaccination and mortality. This study does not establish any such link. The researchers looked only at trends in excess mortality over time, not its causes. The research does not support the claim that vaccines are a major contributory factor to excess deaths since the start of the pandemic. Vaccines have, in fact, been instrumental in reducing the severe illness and death associated with COVID-19 infection.

Update January 2025: BMJ are awaiting the result of an institutional investigation into the conduct of the work, which was due to be finalised by the end of 2024. At present, the institution can offer no update on when the information will be sent to BMJ.
